# 

**Published:** 1883-02

**Authors:** 


THE Mississippi Valley Dental Association, will hold its thirty-ninth annual meeting in Cincinnati at the Ohio Dental College, Wednesday, Thursday and Friday, March 7th, 8th and 9th, 1883.

A full attendance at the opening of the meeting, Wednesday, at ten o'clock, A. M., is desired.

All members of the society and profession are invited.

Friday afternoon will be devoted to a Clinic and the exhibition of appliances.

SUBJECTS FOR DISCUSSION.

1. Nervous and Muscular affections, dependent on Dental Irritation.

2. Etiology and pathology of dental caries. Treatment by filling.

3. Periodontits and alveolar abscess, pathology and treatment.

4. Prosthetic Dentistry: Restoration of features and expression, how best accomplished.

5. Fitting artificial crowns to roots of natural teeth: new and old methods.

6. Reports of cases in practice.

W. L. MOORE, Corresponding Secretary.

### Ohio Dental College Association.

The annual meeting of this body will be held in the lecture room of the College, on Tuesday, March 5th, at 2 o'clock, P. M. It is desirable that all the stockholders, so far as possible, will be present, and take such action as will be for the best interest of the College and the profession.

The Mississippi Valley Dental Society will meet on the morning of the succeeding day. Let all interested be prompt at both of these meetings.

### Transactions.

The volume of Transactions of the last annual meeting of the American Dental Association, has just come to hand. It is well gotten up, both in matter and dress. The publication committee has been rather more prompt than usual, and the work was never better done. This is a valuable volume and should be in the hands of every dentist. It can be obtained of the Secretary, Dr. Geo. H. Cushing, of Chicago.

### DIRECTORY OF DENTAL SOCIETIES.

---

*American Dental Association*—Meets on the 1st Tuesday of August, 1883, at Niagara Falls. Pres. W. H. Goddard, Louisville, Ky.; Cor. Sec. A. W. Harlan, Chicago, Ill.; Rec. Sec. Geo. H. Cushing, Chicago, Ill.

*National Dental Association*—Meets Thursday, in Washington City, Aug. 3, 1883. Pres. Jno. B. Rich, New York; Sec. R. Finley Hunt, Washington, D. C.

### STATE DENTAL SOCIETIES.

*Alabama Dental Association*—Meets annually. Next meeting at Montgomery, on the second Tuesday of April, 1883. Pres. G. M. Rousseau, Montgomery; Sec., T. M. Allen, Eufula.

*California State Dental Association*—Tuesday, June 7th, 1883. Pres. Wm. Dutch, San Francisco; Sec. S. E. Goe., San Francisco; Cor. Sec. H. E. Knox, San Francisco.

*Connecticut State Dental Society*—Pres. E. T. Payne; Rec. Sec. Geo. L. Parmelee; Hartford. Semi-annual meeting in October, 1883, at Hartford.

*Dental Society of the State of Maryland and District of Columbia*—Meets annually. Next meeting at Washington City, on the second Tuesday in October, 1883. Pres. B. F. Coy, of Baltimore: Sec. M. W. Foster. M. D.

*Georgia State Dental Society*—Meets second Tuesday in May, 1883, at Atlanta, Ga. Pres D. Hopps, Savannah; Rec. Sec. G. W. H. Whitaker, Sandersville; Cor. Sec. L. D. Carpenter, Atlanta.

*Illinois State Dental Society*—meets annually. Pres. E. C. Stone, Galesburg; Sec. E. Noyes, Chicago. Next place of meeting, Decatur, second Tuesday in May, 1883.

*Indiana State Dental Society*—Meets next year at Indianapolis, on the last Tuesday of June, 1883. Pres. J. E. Cravens, Indianapolis; Sec. Robert Van Valzah, Terre Haute.

*Iowa State Dental Society*—Meets annually. Next meeting at Iowa City, on the first Tuesday in May, 1883. Pres. A. O. Hunt; Rec. Sec. E. E. Hughes, of Newton; Cor. Sec. I. P. Wilson, Burlington.

*Kentucky State Dental Association*—Meets annually, first Tuesday in June, 1883. Next meeting in Louisville. Pres. Dr. Hooper, Owenton; Sec. E. C. Dunn, Louisville.

*Kansas State Dental Association*—Pres. L. P. Meridith, Sec. J. D. Patterson. Next meeting will be held at Topeka, first Tuesday in May, 1883.

*Maine Dental Society*—Meets in August, at Thomaston. Pres. E. J. Robert, Agusta, Sec. C. E Hussey, Beddeford.

*Massachusetts Dental Society*—Meets semi-annually. Pres. D. B. Ingalls, Clinton, Rec. and Cor. Sec. W. E. Page, Boston.

*Michigan State Dental Association*—Meets annually. Next meeting at Detroit, last Thursday in March, 1883. Pres. A. T. Metcalf, Kalamazoo: Sec. Treas. J. Lathrop, Detroit.

*Mississippi State Dental Association*—Will meet third Tuesday of January, 1883, at Jackson. Pres. J. D. Miles, Vicksburg; Sec. G. W. Rembert, Natchez.

*Minnesota Dental Association*—Meets annually. Meets May 26, 1883, at Winona. Pres. W. F. Lewis, Sec C. M. Bailey, Minneapolis.

*Missouri Dental Association*—Meets annually on the first Tuesday of June, 1883, Next meeting at Sweet Springs. Pres. A. N. Fuller; Sec. W. H. Eames, St. Louis.

*Nebraska State Dental Society*—Meets annually. Next meeting July 23, 1883, at Fremont. Pres. J. W. Chadduck, Nebraska City; Sec. W. F. Roseman, Fremont,

*New Jersey State Dental Society*—Meets annually. Next meeting at Long Branch. on Wednesday, July, 20th 1883. Pres. F. A. Levey, Orange; Sec. C. A. Meeker, Newark.

*New Hampshire Dental Society*—Pres. D. W. Eagerly, Farmington; Sec. C. W. Clerment, Manchester.

*North Carolina State Dental Society*—Meets annually. Next meeting at Raleigh, first Wednesday in September, 1883. Pres. V. E. Turner; Sec. D. A. Robinson.

*Ohio State Dental Society*—Meets annually at Columbus, first Wednesday of October, 1883. Pres. J. W. Lyder, Akron; Sec. J. H. Warner, Columbus.

*Oregon State Dental Society*—Meets annually. Pres. J. R. Cardwell, Portland; Rec. Sec. S. J. Barber.

*Pennsylvania State Dental Society*—Meets Tuesday, July 27th, 1883, at Chatauqua Lake; Pres. H. Gerhart, Lewisburg; Rec. Sec. E. P. Kremer, Lebanon, Pa.; Cor. Sec. E. B. Long, Pittston.

*Rhode Island Dental Association*—Pres. A. W. Buckland, Woonsocket; Sec. L. L. Buckland, Providence.

*South Carolina State Dental Association*—Meets annually. Pres. B. A. Muckenfuss; Sec. G. F. S. Wright, Columbia; Cor. Sec. R. A. Smith. Next meeting at Cheraw, May 3, 1883.

*Texas State Dental Association*—Meets in the city of Austin, May 4, 1883. Pres.——— Sec. W. R. Clifton.

*Tennessee Dental Association* — Meets Annually. Next meeting at Nashville third Tuesday in February, 1883. Pres. J. H. Prewitt, Madisonville, Ky.; Rec. Sec. H. W. Morgan, Nashville; Cor. Sec. J. F. Crawford, Nashville.

*The Dental Society of the State of New York*—Meets annually on the second Wednesday in May. Next session at Albany, May 12, 1883. Pres. C. E. Francis, New York; Rec. Sec. S. A. Freeman, Buffalo; Cor. Sec. W. H. Atkinson, New York.

*Vermont State Dental Society*—Meets annually. Next meeting at Burlington, March 15, 1883. Pres. L. T. Lawton, Rutland, Sec. C. F. Lewis, Burlington.

*Virginia State Dental Association*—Meets in Charlottsville, Tuesday, August 19, 1883. Pres. J. Hall Moore; Sec. L. M. Cowarden, Richmond.

*Wisconsin State Dental Association*—Meets annually. Next meeting at Milwaukee, July 20, 1883. Pres. M. T. Moore, La Crosse; Sec. Geo. H. McCansey, Janesville.

### LOCAL SOCIETIES.

*American Academy of Dental Science*—Meets annually in Boston, Mass., on the last Wednesday in October, 1883. Pres. J. L. Williams; Rec. Sec. Jno. T. Codman; Cor. Sec. C. P. Wilson, Boston.

*Brooklyn Dental Society*—Meets monthly. Pres. Chas. D. Cooke; Cor. Sec. W. H. Atkinson. Rec. Sec. A. P. Crandall, 508 Clinton Ave., Brooklyn.

*Central Dental Association of Northern New Jersey*—Meets annually in February. Pres. C. A. Meeker, Newark, Sec. G. Carleton Brown, Elizabeth.

*Chicago Dental Society.*—Prest., E. S. Talbot; Sec. H. F. Kimball; Cor. Sec. A. W. Harlan.

*Cincinnati Dental Society*—Meets on the second Tuesday in each month. Pres. W. D. Kempton; Sec. A. G. Rose.

*Connecticut Valley Dental Society*—Meets 27 and 28 of Oct. 1883, at Savin Rock Conn. Pres. C. T. Stockwell, Springfield, Mass. Sec. A. M. Ross, Chicopee.

*East Tennessee Dental Association*—Meets annually. Next meeting will be held at Chattanooga, Tenn., on the first Tuesday of Aug., 1883. Pres. W. F. Fowler, Greenville; Sec. W. M. Snyder, Athens.

*New Hampshire State Dental Society*—Meets annually. Pres. H. Hill, Manchester; Sec. E. B. Davis, Concord.

*Merrimack Valley Dental Association*—Meets annually. Pres. J. N. Chandler, Boston; Sec. H. W. Coburn, Lowell, Mass.

*Mississippi Valley Dental Society*—Meets annually at Cincinnati, on first Wednesday in March, 1883. Prest. Dr. J. M. Clyde, Covington, Ky.; Sec. N. S. Hoff, Cincinnati.

*Nashville Dental Association*—Meets on the second Tuesday of each month, Pres. R. R. Freeman; Sec. L. G. Noel, Nashville.

*Northern Ohio Dental Association*—Meets annually. Next meeting at Sandusky, O., on the second Tuesday in May, 1883. Pres. D. Gale French of Pittsburg, Pa.; Rec. Sec. A. J. Douds, Canton; Cor. Sec. H. F. Harvy, Cleveland, O.

*Ohio Dental College Association*—Meets annually, Tuesday, March 2, 1883, at Cincinnati. Pres. H. M. Reid, Cincinnati; Rec. Sec. F. A. Hunter, Cincinnati.

*Odontographic Society of Pennsylvania*—Meets on the first Wednesday of each month, at 8 o'clock p. m., in the Philadelphia Dental College. Pres. J. L. Eisenbrey, Rec. Sec. E. L. Hewitt.

*Odontological Society of New York City*—Meets the second Tuesday of each month. Pres. S. G. Perry; Rec. Sec. Wm Jarvie, Jr.

*Pennsylvania Association of Dental Surgeons.*—Pres. J. H. Githens. Rec. Sec. Theo. F. Chupein.

*Pittsburg Dental Society*—Meets the second Tuesday Evening of each month. Pres. N. W. Aithen; Sec. L. Depriz.

*Fifth District Dental Society of N. Y.*—Meets semi-annually. Next meeting in Syracuse, on the 12th of October, next. Pres. E. L. Swartwout, of Utica. Sec. I. C. Curtis of Fulton.

*San Francisco Dental society*—Meets monthly. Pres. Alex. Warner: Sec. A. W. Knowles; Treas. J. J. Birge.

*Sixth District Dental Society of New York*—Meets semi-annually. Next meeting at Cortland, Thursday, Oct. 6th, 1883. Pres. L. E. Freeland, Onconta; Sec. A. J. Wright, Oswego.

*Washington Dental Society*—Meets second Tuesday of each month. Pres. R. Finley Hunt; Rec. Sec. H. M. Schooly.

*Southern Dental Association*—Meets in Atlanta, Ga., on the second Tuesday of August, 1883. Pres. L. D. Campbell, Atlanta, Ga; Sec. W. H. Hoffman, Charlotte, N. C.

*First District Dental Society of the State of New York*—Meets annually. Pres. A. L. Northrup; Sec. J. E. Dexter, New York.

### EXCHANGES.

St. Louis, Medical and Surgical Journal.
The Pacific Medical and Surgical Journal, San Francisco.
Cincinnati Lancet and Clinic.
Dental Cosmos, Philadelphia.
Chicago Medical Examiner.
The Detroit Review of Medicine and Pharmacy.
Medical Record, N. Y.
American Journal of Dental Science, Baltimore.
Indiana Journal of Medicine.
Missouri Dental Journal, St. Louis.
The Sanitaran, New York City.
L'art Dentaire, Paris.
Le Progress Dentaire, Paris.
Deutische Verteljahrsschrift, Leipzig.
Physician and Surgeon, Ann Arbor, Mich.
Ohio State Journal of Dental Science, Toledo, Ohio.
" " " " " " Xenia, O.
The Microscope, Ann Arbor, Mich.
Scientific American, New York.
Independent Practitioner, N. Y.
The Dental Record, London.
The Journal of the British Dental Association.
The Southern Dental Journal.

### CONTENTS.

COMMUNICATIONS.

### THE DENTAL REGISTER,

A MONTHLY MAGAZINE DEVOTED TO THE INTERESTS OF THE

### DENTAL PROFESSION.

*Terms Reduced to* $2.00 *Per Year.* *Single Copies,* 25 *Cents.*

### EDITED BY PROF. J. TAFT, D.D.S.

—AND—

***Published by SPENCER & CROCKER, Ohio Dental Depot.***

The volume commences in January and closes in December.

The REGISTER being issued monthly is always fresh, therefore Indispensable to the practitioner who desires to keep posted up with the new inventions and improvements which are daily developed. Neither expense nor effort will be spared to give value and variety to its contents. Articles will be illustrated with well executed engravings whenever they will add to the interest or assist to explanation.

Its extensive circulation and frequency of issue make it one of the BEST DENTAL ADVERTISING MEDIUMS in the United States.

All subscriptions must begin with January or July.

Local or special notices, five lines or less, will be inserted for $3 each insertion; over five lines, 50 cts. per line.

All advertising bills payable in advance, or at furthest, after the issue of the first number containing the advertisement. All transient advertisements to be paid for in advance.

☞CONTRIBUTIONS SOLICITED.

Exclusively Editorial Communications should be addressed to the Editor.

The latest number of the REGISTER may be ordered with or without other goods at any time, and all letters should be addressed to

**SPENCER & CROCKER, Ohio Dental Depot, Cincinnati, O.**

